# Olfactory Dysfunction as a Global Biomarker for Sniffing out Alzheimer’s Disease: A Meta-Analysis

**DOI:** 10.3390/bios8020041

**Published:** 2018-04-13

**Authors:** Alisha M. Kotecha, Angelo D. C. Corrêa, Kim M. Fisher, Jo V. Rushworth

**Affiliations:** 1 Faculty of Health and Life Sciences, De Montfort University, Leicester LE1 9BH, UK; alishakotecha@live.co.uk (A.M.K.); kmfisher@dmu.ac.uk (K.M.F.); 2 Department of Diagnostic Radiology, Clementino Fraga Filho University Hospital, Federal University of Rio de Janeiro, Rio de Janeiro 21941-901, Brazil; dante.angelo@gmail.com

**Keywords:** Alzheimer’s disease (AD), mild cognitive impairment (MCI), biomarker, dementia, olfactory dysfunction, olfaction, smell

## Abstract

Cases of Alzheimer’s disease (AD) are rising exponentially due to increasing global life expectancy. There are approximately 50 million sufferers worldwide, with prevalence rising most rapidly in low-income countries such as Africa and Asia. There is currently no definite diagnosis of AD until after death, thus an early biomarker for AD is urgently required in order to administer timelier and more effective interventions. Olfactory dysfunction (problems with the sense of smell) is one of the earliest, preclinical symptoms observed in AD. Olfaction is a promising early biomarker for use worldwide as it is easy, cheap to measure, and not reliant on specialist clinicians or laboratory analysis. We carried out a meta-analysis to determine the credibility of olfaction in diagnosing AD in the preclinical stages, by comparing olfaction in healthy controls against AD patients and patients with mild cognitive impairment (MCI). Data from 10 articles were subjected to two comparative meta-analyses. In the case of AD, the results illustrated that the overall magnitude of effect size was more apparent, *d* = −1.63, 95% CI [−1.95, −1.31], in comparison to that of MCI, *d* = −0.81, 95% CI [−1.08, −0.55]. This shows that olfaction worsens progressively as patients progress from MCI to AD, highlighting the potential for olfactory dysfunction to identify AD in the preclinical stages prior to MCI.

## 1. Introduction

**Figure 1:**
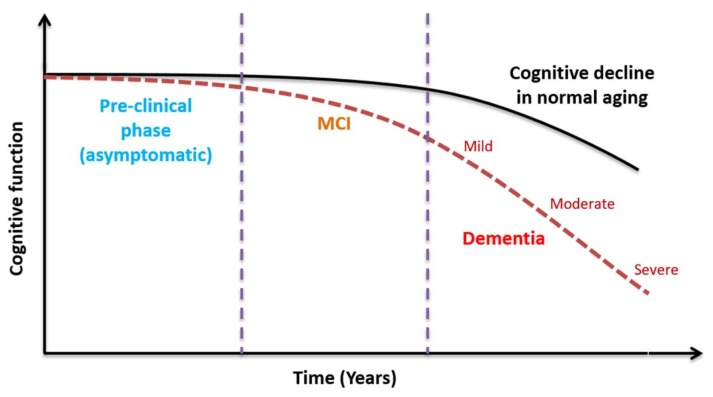
The decline in cognitive function in the presence of Alzheimer’s disease (AD) is much more rapid than, and distinct from, cognitive decline seen in normal aging. A preclinical biomarker is required to distinguish the AD pathway from the normal aging pathway, allowing timely therapeutic intervention.

Alzheimer’s disease (AD) is the most common cause of dementia. AD is classified as a critical worldwide public health issue due to its significant health, medical, and socioeconomic burden on society. Globally, AD is thought to affect over 47 million people at a substantial cost of over $818 billion per year [1]. Cases of AD are rising exponentially due to the increasing life expectancy of the human population [2,3]. Currently, quantifying the sociological and psychological burden of the care and management of AD patients remains difficult and can also incur a substantial financial cost [4]. There is currently no cure for AD, due to the mechanisms of disease remaining complex and not understood fully. Whilst there is medication that can alleviate the symptoms of AD, the lack of a robust and early diagnosis means that intervention is currently given too late to be effective. An early biomarker for AD is required urgently. If the ability to diagnose AD during the presymptomatic stages (Figure 1) becomes more reliable, then quicker more successful therapeutic and disease monitoring methods can be achieved [5]. Developing countries such as India and China, with rapidly expanding populations and increasing life expectancies, are seeing the biggest rise in AD cases [6]. Accordingly, a useful global biomarker for AD needs to be cheap, simple to evaluate and non-reliant upon specialist clinical or scientific analysis.

AD brain pathology indicates that the hippocampus (memory centre) of the brain is first affected. In the later stages, AD progressively affects the cortex and other brain regions for higher brain function controlling thoughts, actions, and personality [7]. The rate in which AD can develop varies from one individual to another. Mild cognitive impairment (MCI) is first observed, which gradually impedes daily life as the condition progresses to AD. Memory loss, personality changes, and difficultly performing everyday tasks become more pronounced as the disease progresses. Patients will also display behavioural changes and ultimately have difficulty in communicating, feeding, and swallowing [8,9]. Presently, there are no independent methods for diagnosing AD; in many cases, physicians and neurologists undertake an extensive and comprehensive medical history of the patient, including conducting cognitive, physical, and neurological examinations and imaging. The Mini-Mental State Examination (MMSE) is commonly used to estimate the severity and progression of cognitive impairment and to provide a diagnosis of dementia [10]. However, AD shares many neuropathological features with other diseases, meaning that a definitive diagnosis is only achievable through clinical assessment and post-mortem verification of the hallmarks of disease in the brain [11].

A biological marker or biomarker can be objectively measured to accurately and reliably diagnose or envisage a physiologic or pathologic condition [12]. In the case of AD, a biomarker able to detect the disease in the early clinical stages would facilitate earlier diagnosis and disease management. A global biomarker for AD should be cheap, specific, sensitive, reliable, and not reliant upon specialist clinicians or laboratory testing [13]. Cerebrospinal fluid (CSF) is thought to be the optimum source for biomarker development in neurological diseases such as AD [14,15]. This is because it is in direct contact with the brain and, through its molecular composition, it can show biological modifications within the brain. However, CSF collection is an invasive procedure and its method to collect the fluid is not widely employed because it cannot be used as part of follow-up analysis of the patient. There is now more promise from blood-based biomarkers as they are less invasive, easily accessible, and are more cost effective in comparison to CSF and imaging biomarkers. The two main issues faced with these methods are that trained clinicians are still required and that there is an increased diagnosis period.

**Figure 2:**
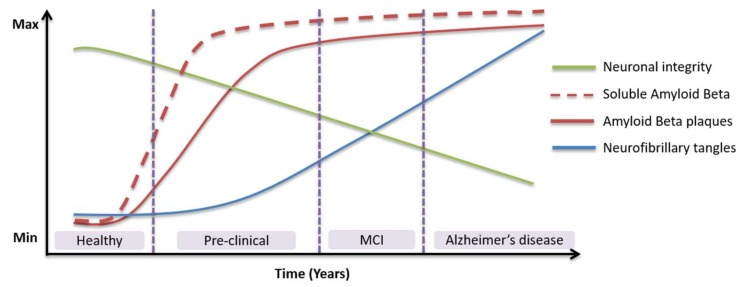
Alterations in levels of established biomarkers in relation to the neuropathology and clinical changes of Alzheimer’s disease.

The two key pathological hallmarks of AD are extracellular senile plaques of amyloid-beta (Aβ) and intracellular neurofibrillary tangles of hyperphosphorylated tau protein [16,17]. Aβ peptides undergo misfolding and aggregation to form toxic soluble oligomers (AβOs) of varying sizes and shapes [18]. Levels of AβOs in the brain have been shown to correlate with AD onset and severity and have therefore been proposed as a biomarker for AD [19,20,21]. Tau is an intracellular protein that usually stabilises microtubules, which are crucial for axonal transport and signal transduction [22]. In AD, tau is destabilised by aberrant hyperphosphorylation, detaches from the microtubules, and forms cytotoxic neurofibrillary tangles [23]. These proteins cause oxidative stress within the cytosol and lead ultimately to cell death [24]. Aβ, total tau (T-tau), and phosphorylated tau (P-tau) are well-established CSF or blood-based biomarkers for AD [25]. Changes in the levels of Aβ and tau within an individual over the lifetime may be a theoretical indicator of AD (Figure 2). However, these species can also be elevated in other disorders, therefore, novel biomarkers are required urgently [26].

Various novel blood- and CSF-based biomarkers for AD have been proposed in recent years. These include (1) neuroinflammatory markers such as reactive oxygen species (ROS), cytokines, chemokines, astrocytes, and activated microglia; (2) proinflammatory molecules such as interleukins (ILs), interferons (IFNs), and tumour necrosis factors (TNFs); (3) autoantibodies; (4) trace elements such as copper, zinc, iron; (5) fatty acids, sphingolipids, ceramides; (6) micro-RNAs; (7) circulating nanocomponents; and others [26,27,28,29,30,31]. Alternatively, non-invasive neuroimaging techniques such as computerized tomography (CT), magnetic resonance imaging (MRI), and positron emission tomography (PET) offer structural and functional information about the brain, which may be able to support a diagnosis of dementia, but only in the later stages when brain shrinkage is evident. All of these biological and imaging biomarkers are unsuitable global biomarkers for AD, as they require expensive equipment and trained clinicians to perform the procedure and to subsequently interpret the findings [32].

**Figure 3:**
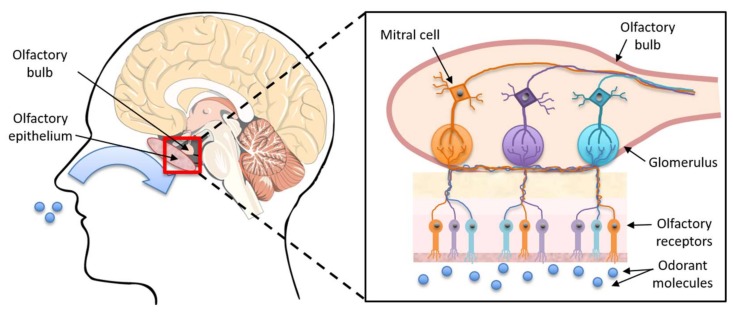
The olfactory system.

Olfactory dysfunction is one of the earliest symptoms of AD, highlighting its potential as a biomarker for early, preclinical diagnosis [33]. The olfactory system provides our sense of smell and consists of sensory neurons found in the olfactory epithelium (Figure 3), which thins during normal aging [34]. The neurons are ciliated bipolar cells with a dendritic ending and with cilia projecting into the mucus. Unmyelinated axons form into bundles, called the olfactory fila, and protrude through the cribriform plate to the olfactory bulb. This triggers a signal transduction pathway and the sequential depolarization of axons upon receiving an olfactory stimulus. Upon the signal reaching the olfactory bulb, olfactory neurons with complementary receptors bind and synapse with secondary-order (mitral and tufted cells) neurons forming glomeruli. The second-order neurons are extensions off the olfactory bulb leading to the olfactory tract and the primary olfactory cortex areas, which lie in close proximity to the hippocampus which is the primary site of early AD pathology [24].

The pathology of AD impacts numerous parts of the brain where the olfactory system functions. Anatomical-pathological changes are present in the key areas for odour recognition and memory including the peripheral and central olfactory cortex, olfactory bulb and tract regions, and layers II and III of the entorhinal cortex and the horn of Ammon. Deficits in the main olfactory domains (odour detection, recognition, and discrimination) have been reported to appear before cognitive impairment in AD. Multiple studies have shown olfactory loss, hyposmia (reduced ability to detect and smell odour), and anosmia (the complete loss of odour detection and sense of smell) are a part of aging. One of the main questions to be addressed is whether the biomarker can be used accurately to differentiate between age-related olfactory reduction and disease-induced dysfunction. Although the neuropathological pathway leading to olfactory dysfunction has not been elucidated, research has exhaustively demonstrated lesions such as senile plaques and neurofibrillary degeneration throughout the olfactory system, which appears to be the most supported hypothesis [34].

There are three commonly used smell tests within scientific research: the University of Pennsylvania Smell Identification Test (UPSIT), the Brief Smell Identification Test (B-SIT), and the Sniffin’ Sticks Test (SST) [35,36,37,38]. The UPSIT is a “gold standard”, comprehensive 40-item test that can be self-administered. It is considered the most reliable and accurate olfactory test available. The odour is embedded within microencapsulated crystal pads, which is released upon the subsequent scratching of the impregnated test booklets using a pencil tip. Physically, it involves four booklets with 10 odorants on each page, which must be identified by the participant from a multiple of four choices. The B-SIT is essentially a shorter version of the UPSIT consisting of 12 items, allowing the test to be performed rapidly. This version of the test is employed in instances where time is short, for example, in less economically developed countries where clinicians are overstretched. SST is a nasal chemosensory performance test. It combines three subtests of olfactory function, using an odour pen-like administration device, to measure odour identification, threshold, and discrimination.

Here, a meta-analysis was carried out to determine if olfactory dysfunction is a potential biomarker for Alzheimer disease, and to determine its credibility of diagnosing AD in the preclinical stages. Our findings indicate that olfactory function declines as patients progress from MCI to a diagnosis of AD, hence presenting the ability of olfaction to identify AD in the preclinical stages prior to MCI.

## 2. Materials and Methods

### 2.1. Identification of Studies to Include in the Meta-Analysis

**Figure 4:**
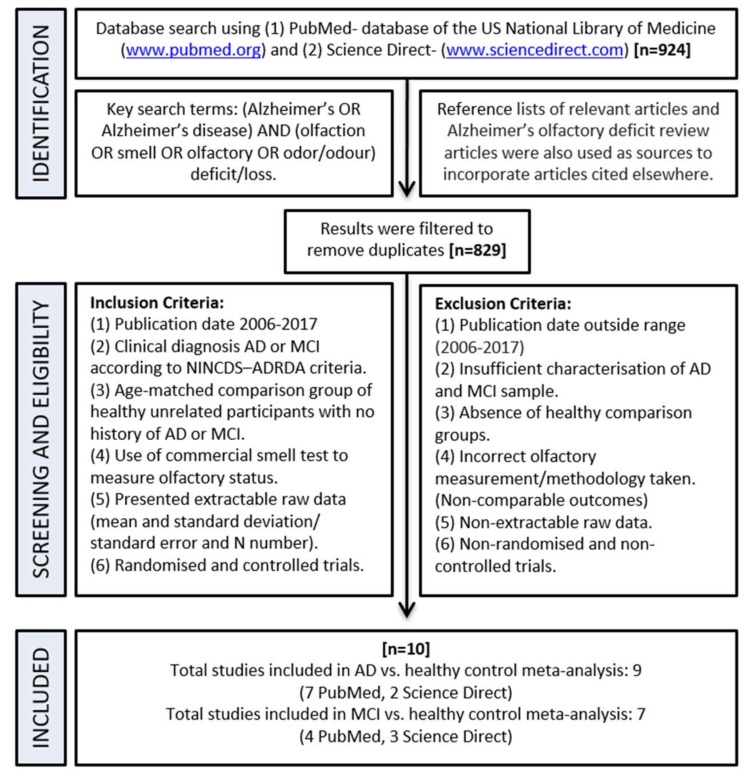
A flow chart showing the process of identifying suitable studies for the meta-analysis.

A search, filter, and exclusion strategy was carried out as indicted by the schematic flow chart in Figure 4. Initially, 924 articles were identified in the search step, which were screened and filtered to leave 10 studies which met the inclusion and exclusion criteria.

### 2.2. Meta-Analysis

Data were entered into Review Manager (RevMan) version 5.3.5 (The Cochrane Collaboration, Copenhagen, Denmark) for meta-analysis [39]. The method of data entering used was for continuous data in which there was raw data for each study. Random-effect models were used to calculate the significance level of the mean effect sizes for each study. The analysis was carried out using standardised mean differences (SMD), as although all of the olfaction tests ultimately measure the same outcome, their absolute values cannot be combined. A funnel plot of standard error against standardised mean difference was generated to enable a visual investigation into possible publication bias.

## 3. Results

### 3.1. Characteristics of Included Studies

Initially, 924 articles were identified from the initial PubMed and Science Direct database search, which were then screened and filtered to remove duplicates. All of these articles were assessed for eligibility according to set criteria (Figure 4) and eventually 10 were included in the meta-analyses (Table 1). To summarise these 10 articles, 7 came from PubMed and 3 from Science Direct.

**Figure 5:**
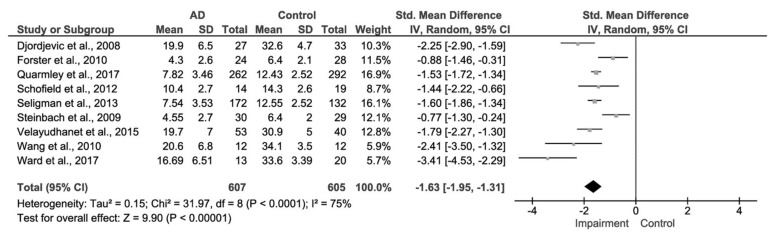
Forest plot comparing odour identification ability between AD and healthy control patients.

Six of the 10 studies had data regarding all three patient groups (AD, MCI, and control patients), 3 with just AD and control group and 1 with MCI and control groups (Figure 5). These studies were conducted from 2008 to 2017 and showed conclusions that were largely heterogeneous. The heterogeneous conclusions of these studies were because of inclusion criteria, experimental design, and the type and number of olfactory tests conducted. A range of different olfactory tests were carried out across these studies, the most common being the UPSIT. The UPSIT tests ranged from 12 to 40 items dependent upon the study. All of the included studies used National Institute of Neurological and Communicative Disorders and Stroke and the Alzheimer’s disease and Related Disorders (NINCDS-ADRDA) diagnostic criteria, and two studies also used the DSM-IV criteria. Sample sizes varied across studies and extended from as small as 8 people to as large as 292.

### 3.2. AD and Control Group

Data were initially meta-analysed for the olfactory function in diagnosed AD patients in comparison to a healthy control cohort (Figure 5). Of the eligible studies, nine reported extractable data on both AD patients and healthy comparison control groups. These studies comprised 607 patients with AD and 605 control patients in total. Again, the publication date ranged from 2008 to 2017. The mean age of AD patients ranged from approximately 70 to 78 years, thus displaying across all the studies that there was a narrow age range. Out of the included studies, four used the SSIT and five used the UPSIT.

The meta-analytic summary indicated a significant difference in olfactory function of odour identification between AD patients and control patients of −1.63, 95% CI [−1.95, −1.31], *p* < 0.00001.

### 3.3. Mild Cognitive Impairment (MCI) and Control Group

**Figure 6:**
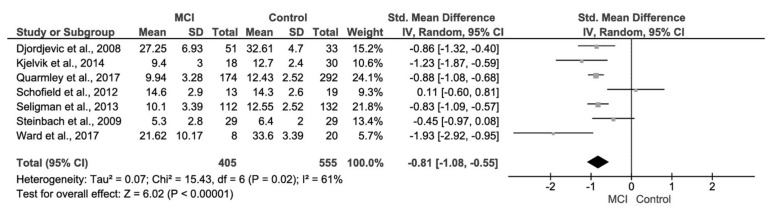
Forest plot comparing odour identification ability between MCI and healthy control patients.

Fewer studies were available for the meta-analysis of MCI patients in comparison to a healthy control cohort (Figure 6). Of the eligible studies, only seven reported extractable data on both MCI patients and healthy comparison control groups, in which the majority (six studies) of these studies also evaluated AD patients as well as MCI. These studies comprised a total of 555 control patients and 405 patients with MCI. The MCI patients had a mean age ranged from approximately 65 to 78 years, also showing a narrow age range.

The meta-analytic summary indicated a significant difference in olfactory function of odour identification between MCI patients and control patients; *d* = −0.81, 95% CI [−1.08, −0.55], *p* < 0.00001.

### 3.4. Comparison of Effect Sizes and Test of Publication Bias

**Figure 7:**
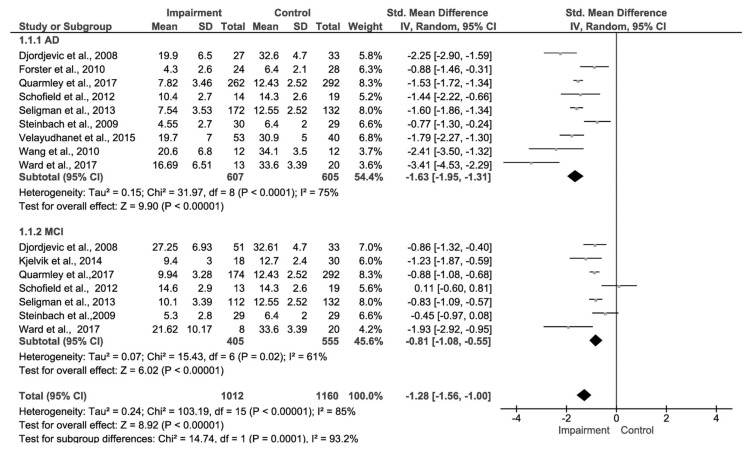
Subgroup analysis forest plot comparing the effect sizes of the two meta-analyses conducted.

**Figure 8:**
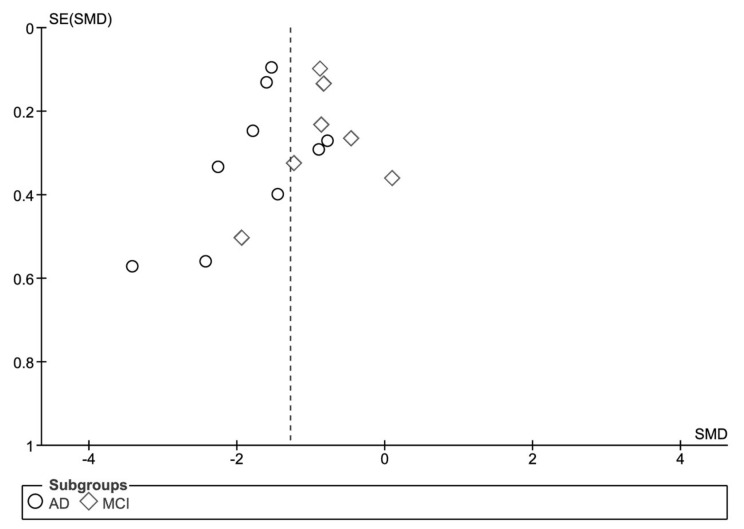
Funnel plot analysis.

A forest plot was generated to compare the effect sizes of the two meta-analyses undertaken (Figure 7). The effect size between the two subgroups is significantly different (Chi^2^ = 14.74, *df* = 1 (*p* = 0.0001), *I*^2^ = 93.2%). Funnel plot analysis was undertaken to evaluate visually the presence of publication bias (Figure 8). The funnel plot for the MCI data is generally symmetrical which indicates the absence of publication bias, suggesting that the published studies used in the meta-analyses are a representative sample of the available evidence. There is a little asymmetry in the case of the AD funnel plot with two of the studies with smaller sample sizes [44,49], providing a larger effect size than other studies in this comparison. However, these two publications contribute a small weighting to the study and their exclusion would not alter the overall conclusion.

The standard errors were plotted against the standardised mean differences for the MCI studies (diamonds) and the AD studies (circles).

## 4. Discussion

This meta-analysis extends the current literature on olfactory dysfunction in AD and MCI patients by quantifying the scale of known olfactory deficits, comparative to healthy control patients. Notably, the results obtained from the meta-analysis illustrate that in the case of AD the overall magnitude of effect size was relatively large (*d* = −1.63, 95% CI [−1.95, −1.31]) in comparison to that of MCI (*d* = −0.81, 95% CI [−1.08, −0.55]). Although the olfactory deficit is statistically significant in both AD and MCI patients, it differs to a greater extent between AD and control patients due to the higher effect size value. This suggests that olfactory function is a progressively diminishing factor as a patient goes from MCI to being diagnosed with AD, which suggests that detecting a small decline in an individual’s sense of smell may be able to be exploited as a very early, preclinical indicator of AD.

However, both meta-analyses demonstrate high heterogeneity with an *I*^2^ value of 75% for AD vs. control and 61% for MCI vs. control. This may be due to clinical heterogeneity (variation in participants, interventions, and outcomes), or methodological diversity (variability in study design and risk of bias). Future work to reduce heterogeneity and to validate the outcome of this research could involve obtaining additional, unpublished information about the patients contained within the studies examined here. This unpublished information could contain data such as gender distribution, smoking status, and patients’ medical history, which are potentially influencing factors that could be considered to affect heterogeneity. Furthermore, conducting sub-meta-analysis by subgrouping the studies according to their differences, and carrying out smaller meta-analysis on more similar characteristics could also theoretically reduce heterogeneity.

Our results are congruent with previous studies that have shown AD patients to have a greater olfactory impairment. In a recent meta-analysis of 39 studies, a large effect size (Cohen’s *d* = 1.73) was reported for AD [50]. The review concluded that AD has a significant effect on olfactory ability. It predominantly showed odour identification, recognition, and other tests that assess these olfactory measurements could be beneficial if included in a battery of exams in order to detect subclinical AD. A systematic review conducted by Sun and co-workers evaluating the utility of olfactory identification tests as prognostic tools for AD, through 30 cross-sectional studies and two potential longitudinal cohort studies, presented a large body of evidence and established a link between decreased olfaction (hyposmia) and AD [51]. The study showed the potential value of olfactory tests as predictors for the onset of AD and also in prognosis; however, a meticulously designed longitudinal cohort of studies is required to authenticate these findings. In comparison, Roalf and colleagues stated a moderately lower Cohen’s *d* = 0.76 for MCI, and concluded that odour identification is the most extensively impaired in MCI, due to generating a larger effect size compared to those of odour detection, threshold, or memory [52]. The effect values from both these studies correlate well with this study’s presented findings, in spite of a broad study inclusion date in the case of [50] and the inclusion of homemade olfactory test in [52] which does not produce consistently comparable results. Reduced olfactory identification (OI) was shown recently to be associated with AD pathology in healthy individuals, although the correlation between OI and cognitive decline was variable [53].

AD pathology in the entorhinal cortex, hippocampus, and temporal regions of the brain is believed to underpin the inability to store and recollect memories of smell, explaining why AD sufferers cannot correctly identify odours presented to them [54]. The underlying mechanisms of olfactory dysfunction and how these relate to AD pathology is not elucidated fully. Evidence of both neurofibrillary and amyloid-β deposition has been seen in AD rodent models and has been indicative of olfactory dysfunction [55,56]. Mouse model studies suggest that olfactory deficits in AD are linked to the presence of AβOs within the first synaptic processing stage of the olfactory system [56]. Interestingly, the cellular prion protein (PrP^C^), a recently identified receptor which mediates the toxicity of AβOs, is highly expressed in the olfactory bulb as well as the hippocampus [19,57]. The olfactory bulb has recently been proposed as the site of entry for prion-like propagation in neurodegeneration [58]. The link to memory impairment and hence inability of odour identification is also postulated to be related to degeneration of neurotransmitters such as acetylcholine, glutamate, and γ-aminobutyric acid, which are normally abundant in the brain and are involved in olfactory signal-transduction [54].

Although olfaction is a promising biomarker for AD, it has also been identified as a potential indicator of other neurodegenerative diseases including Parkinson’s disease (PD) [59], Huntington’s disease [60], multiple sclerosis (MS) [61], and amyotrophic lateral sclerosis (ALS) [62]. Therefore, future work in this area needs to focus upon improving the specificity of olfaction as a biomarker of AD. One previous meta-analysis revealed that the olfactory deficits observed in AD and PD could not be distinguished [63]. A more recent meta-analysis by Rahayel and colleagues revealed that, whilst AD and PD patients both demonstrate impairments in odour recognition and identification, PD patients are more impaired in odour detection thresholds [50]. Furthermore, the effect sizes found in the AD studies were greater than the PD studies, compared to control. Olfactory tests using particular subsets of odorants (i.e., particular smells) may be able to identify specific neurodegenerative disorders, although this requires standardisation and further optimisation [64]. Further investigation into whether different domains of olfaction (odour identification, recognition, or discrimination) are impaired to different extents in different diseases is also required. For example, odour threshold scores were found to be altered in MS versus control patients, whereas other olfactory parameters were unaltered [61]. In AD, odour identification appears to be the most reliable indicator of disease [52,53]. Hummel and colleagues proposed recently that patients displaying olfactory dysfunction should undergo a battery of investigations, emphasising that olfactory assessment should not be undertaken in isolation due to its poor reliability and links with a multitude of disorders [65].

## 5. Conclusions

The evidence presented suggests that olfactory dysfunction is more commonly shown to affect odour identification most extensively and occurs within the initial stages of the AD disease process where the patient shows MCI. It also suggests that the dysfunction occurs potentially prior to this in the preclinical stages and hence can potentially be superior to other conventional AD biomarkers, due to the ease of administration, being relatively inexpensive, and non-invasive. This allows people to benefit from first interventions to avert or modulate risk of progression. Although, without further development of longitudinal studies with large cohorts, olfactory testing can only be used as a screening tool along with cognitive, physical, and neurological examinations (MMSE) and imaging in order to enhance diagnostic sensitivity and specificity. To progress this work further, longitudinal analyses studies looking at AD and MCI (and the varied subtypes) patient groups over a period of time would clarify and enhance the findings due to uniform methodology and olfactory testing. This would further improve the development of more successful therapeutic intervention and disease monitoring. However, due to the multifactorial nature of AD, both neurologically and clinically, the prospect of being able to produce a single biomarker that will sufficiently meet the needs for clinical diagnosis is unlikely at this stage. Focuses should be shifted towards the standardisation of a panel of biomarkers (including olfactory testing) that would offer the sensitivity and specificity required for diagnosing AD.

## Figures and Tables

**Table 1:** Characteristics of the 10 studies included in the meta-analyses.

Study Citation	Smell Test	Mean Age (±SD/Range)			Sample Size			Mean Score		
		AD	MCI	CTRL	AD	MCI	CTRL	AD	MCI	CTRL
Steinbach et al., 2010 [40]	SSIT	73.3 ± 7.8	71.7± 7.7	68.2 ± 3.9	30	29	29	4.55 ± 2.7	5.3 ± 2.8	6.4 ± 2.0
Schofield et al., 2012 [41]	UPSIT-20	75.3 ± 4.6	77.1 ± 5.6	74 ± 6.6	14	13	19	10.4 ± 2.7	14.6 ± 2.9	14.3 ± 2.6
Djordjevic et al., 2008 [42]	UPSIT-40	77.0 (55–88)	75.4 (59–86)	73.7 (63–87)	27	51	33	19.89 ± 6.5	27.25 ± 6.93	32.61 ± 4.7
Kjelvik et al., 2014 [43]	B-SIT-12	-	67.4 ± 7.6	74.6 ± 6.3	-	18	30	-	6.6 ± 2.6	9.6 ± 2.0
	SSIT-16								9.4 ± 3.0	12.7 ± 2.4
	SSDT-16								7.5 ± 3.0	9.2 ± 3.3
Wang et al., 2010 [44]	UPSIT-40	74.5 ± 7.5	-	67.8 ± 9.8	12	-	12	20.6 ± 6.8	-	34.1 ± 3.5
Quarmley et al., 2017 [45]	SSIT-16	75.18 ± 8.22	72.46 ± 8.57	70.96 ± 8.74	262	174	292	7.82 ± 3.46	9.94 ± 3.28	12.43 ± 2.52
Seligman et al., 2013 [46]	SSIT-16	75.98 ± 7.53	72.63 ± 8.19	72.57 ± 9.52	172	112	132	7.54 ± 3.53	10.10 ± 3 .39	12.55 ± 2.52
Velayudhan et al., 2015 [47]	UPSIT-40	73.5 ± 11	-	70.5 ± 9	54	-	40	19.7 ± 7	-	30.9 ± 5
	UPSIT-12							5.4 ± 3		10.4 ± 2
Förster et al., 2010 [48]	SSIT-12	71.4 ± 7.9	-	68.2 ± 3.9	24	-	28	4.3 ± 2.6	-	6.4 ± 2.1
Ward et al., 2017 [49]	UPSIT-40	76.77 ± 6.95	76.13 ± 6.29	76.65 ± 6.48	13	8	20	16.69 ± 6.51	21.62 ± 10.2	33.60 ± 3.34

Abbreviations: B-SIT: Brief Smell Identification Test; SSDT: Sniffin’ Sticks Discrimination Test; SSIT: Sniffin’ Sticks Identification Test; UPSIT: University of Pennsylvania Smell Identification Test.
